# TRENDS IN HEALTH CONDITIONS, HEALTH NEEDS, HEALTH SERVICES & MEDICATION USE AMONG OREGON AFH RESIDENTS (2017-2023)

**DOI:** 10.1093/geroni/igae098.4385

**Published:** 2024-12-31

**Authors:** Wafi Albalawi, Ozcan Tunalilar, Paula Carder

**Affiliations:** Oregon Health and Science University, Portland, Oregon, United States; Portland State University, Portland, Oregon, United States; OHSU-PSU School of Public Health, Portland, Oregon, United States

## Abstract

The aging U.S. population is increasing the demand for Long-Term Care Services. Adult Foster Homes (AFHs) offer a home-like environment for residents with complex health needs, particularly beneficial for older adults, including those with dementia. However, these settings remain understudied. This study evaluated trends in resident characteristics, chronic conditions, service utilization, medication use, activities of daily living (ADLs), and family involvement among AFH residents in Oregon from 2017 to 2023, using data from the Oregon Community-Based Care study. Data were analyzed using descriptive statistics and OLS regression. Results indicate that the proportion of female residents remained stable at 60-64%. Residents aged 75-84 increased from 18.7% to 25.7%, while those aged 85+ decreased from 41.6% to 31.5%. Hypertension (48.7-51.6%) and dementia (43.6-47.7%) are the most prevalent conditions. Arthritis declined from 37.8% to 30.0%, and multiple medication use peaked at 61.5% in 2023. Bathing (78.4-82.9%) and dressing (60.5-69.5%) are the ADLs requiring the most assistance. Family involvement saw significant changes during the pandemic, with social visits decreasing from 64.9% to 37.8% and outings from 39.8% to 17.4%. The study found both similarities and differences between AFH residents and assisted living (AL) residents in the U.S. Hypertension and dementia were the most prevalent conditions in both groups. However, AFH residents had higher rates of dementia and arthritis, as well as greater reliance on Medicaid (54%-63.2%) compared to AL residents (17%). These findings highlight the higher acuity among AFH residents and emphasize the need for further research on smaller residential settings for older adults.

